# Editorial: Artificial intelligence: applications in clinical medicine

**DOI:** 10.3389/fmedt.2023.1206969

**Published:** 2023-06-09

**Authors:** Joshua Levy, Emilio Madrigal, Louis Vaickus

**Affiliations:** ^1^ Emerging Diagnostic and Investigative Technologies, Department of Pathology and Laboratory Medicine, Dartmouth Hitchcock Medical Center, Lebanon, NH, United States; ^2^ Department of Dermatology, Dartmouth Hitchcock Medical Center, Lebanon, NH, United States; ^3^ Department of Epidemiology, Dartmouth College Geisel School of Medicine, Hanover, NH, United States; ^4^ Program in Quantitative Biomedical Sciences, Dartmouth College Geisel School of Medicine, Hanover, NH, United States; ^5^ Department of Pathology, Massachusetts General Hospital, Boston, MA, United States

**Keywords:** implementation, education, machine learning, medicine, artificial intelligence, stakeholder

**Editorial on the Research Topic** Artificial intelligence: applications in clinical medicine

Exciting opportunities abound for exploring the potential of artificial intelligence (AI) technologies in medicine. The interest in AI has grown substantially across all industries, including healthcare, in part owing to the advent of popular and highly publicized AI technologies. For instance, the emergence of ChatGPT has enabled rapid access to knowledge and information in a highly digestible format (despite its tendency to generate nonsensical responses) (1). The rise of these new technologies has created a lot of enthusiasm and anticipation about the potential of AI to revolutionize healthcare by functioning as an unbiased observer capable of efficiently processing large, intricate datasets. This eagerness can sometimes overshadow practical considerations for the translation of AI from development into clinical implementation. While it is tempting to apply AI to every clinical problem, it should be noted that not every problem can be solved more efficiently with AI technologies. In comparison to ChatGPT, which has already provided users with a series of useful “productivity hacks”, medical AI technologies have not yet delivered on their promises to transform healthcare. This has led to a growing viewpoint that these technologies are in a “trough of disillusionment”—initial excitement and expectations have not yet been met, giving way to increasing skepticism.

To move from the “trough of disillusionment” to the “plateau of productivity” in medical AI technology (Figure 1), it is important to address practical considerations for development and implementation before investing in multicenter clinical trials. This requires a broader set of tools to understand the suitability of technologies for clinical trials and identify barriers to adoption. Implementation science frameworks, such as the Consolidated Framework for Implementation Research (CFIR), can promote the adoption of evidence-based practices and consider additional context beyond the intervention's effectiveness (2–4). Transdisciplinary teams are essential to realizing the potential of medical AI technologies, as not all computer science experts are implementation scientists or clinicians.

**Figure 1:**
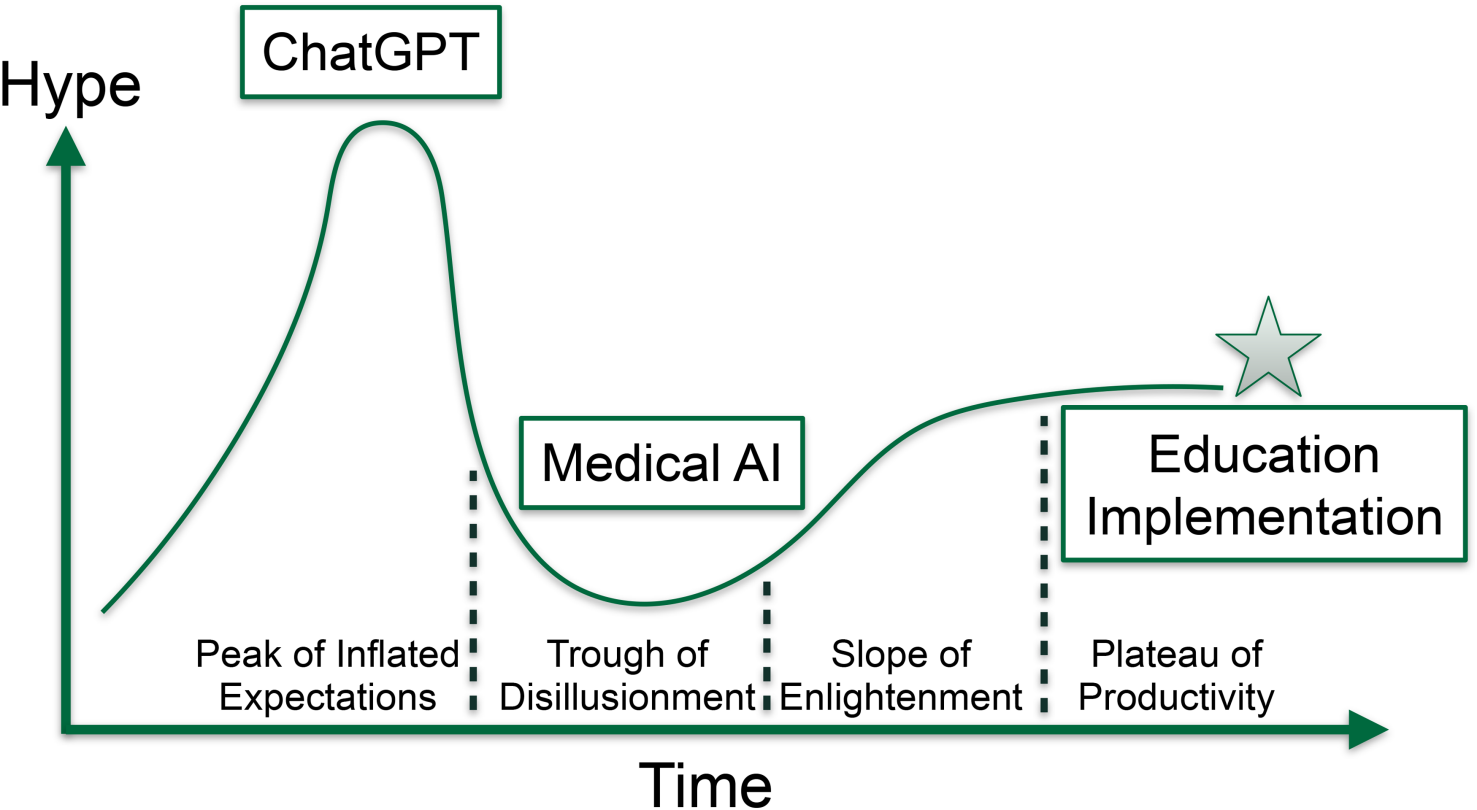
Illustration of the hype curve for emerging medical AI technologies, demonstrating the importance of implementation, education, and training in progressing from the trough of disillusionment to the plateau of productivity.

The development of AI technologies can lead to bias if not approached with a blend of perspectives, values, experiences, and viewpoints. This bias can result in algorithmic behavior that negatively impacts historically underserved groups and can lead to higher under-diagnosis and misdiagnosis rates in these populations. For example, skin cancer image prediction algorithms have been found to be less accurate for underprivileged demographics (5). As AI algorithms become more widely used in clinical care, it is crucial to broaden participation in their development to be more representative of varied populations.

Previous research has shown that highly diverse research groups outperform homogenous groups (6), making it essential to involve practitioners and participants from underserved populations in AI development. Remote learning frameworks present an opportunity to expose students from underrepresented groups to AI research and STEM career pathways where transportation and access to opportunities is limited. Still, there is a need to develop a deeper understanding of the instructional, curricular, and infrastructure adaptations required to successfully implement fully remote research experiences for students of underserved backgrounds (7, 8). This includes providing access to mentors as role models to help them find and leverage resources that can advance career prospects.

We requested articles that adhered to implementation and educational principles for designing, validating, and implementing emerging medical AI technologies. The articles featured include two reviews, an education curriculum design article, and an original research article that broadly covers advances in orthopedics, drug delivery and design, radiology, and margin assessment.

Farhadi et al. summarized current advances and applications of machine learning algorithms across five key orthopedic disciplines, namely, joint reconstruction, spine, orthopedic oncology, trauma, and sports medicine. They noted the lack of adoption of these technologies for clinical use and suggested metrics such as reduction in interpretation time, lower misinterpretation rates, patient outcomes, and complications to communicate the scope and impact of these technologies. The authors also highlighted the importance of utilizing interpretable models capable of recapitulating diagnostic uncertainty and exploring procedures for facilitating rapid data acquisition across multiple institutional partners.

Kaushik et al. explored areas where AI technologies could be used to facilitate treatment planning for cancer patients by mining genomics data to measure and optimize material properties of the therapeutic and selecting optimal therapies based on interactions with the immune system (Das and Chandra). Reinforcement learning, a machine learning approach that operates on feedback from the environment or system of interaction given the selection of a potential solution (i.e., drug design and delivery space), was discussed as a potential tool to predict a sequence of future actions to take based on the patient's state and expected rewards. While there are only sparse applications of reinforcement learning in the biomedical space, real-time feedback on drug discovery/delivery is one domain where these algorithms are being applied for future pharmacogenomic research.

Chen et al. explored the application of machine learning methods to a radiomics-based margin assessment of fluorescence-based paired-agent imaging. Paired-agent images improve the discrimination of tumors based on EGFR targets by the presurgical administration of ABY-029, offering significant advantages beyond standard histological assessment. Complementing improved imaging modalities with image analysis workflows can improve the accuracy, efficiency, and completeness of real-time intraoperative tumor margin assessment. Technologies that enhance the efficiency of the surgical pathology workflow have high likelihood for adoption.

Medical students and residents are entering a rapidly changing healthcare landscape where technology and data will play a crucial role. It will be essential for practicing professionals to gain familiarity with machine learning tools and how to operate them. Having a quantitative skillset paired with clinical domain expertise is crucial for developing technologies germane to work in the clinic. Lindqwister et al. epitomized these aspirations through a longitudinal education curriculum for radiology residents and demonstrated high satisfaction and perceived understanding of AI concepts from a 7-month course and workshop offerings. Considering how AI and medical informatics can be seamlessly integrated into the medical student/resident curriculum is an ongoing debate as trainees are highly pressed for time with competing demands.

In summary, addressing practical considerations for medical AI technology development and implementation, involving diverse perspectives in the development process, and providing access to mentorship and resources for students from underserved backgrounds are crucial steps to move towards the “plateau of productivity” and realize the potential of medical AI technology.

## Author's note

Beyond Hype: The Key Role of Implementation and Education in Realizing the Promise of Emerging Medical AI Technologies. Disclosure: During the preparation of this work the authors applied large language models to perform minor edits to originally drafted content to improve readability and language. After using this tool, the authors reviewed and edited the content as needed and take full responsibility for the content of the publication.
